# Association Between Mothers' Problematic Internet Use and the Thinness of Their Children

**DOI:** 10.1089/cyber.2018.0685

**Published:** 2019-09-17

**Authors:** Aya Sakakihara, Chiyori Haga, Yoneatsu Osaki

**Affiliations:** ^1^ Community Health Nursing, Faculty of Medicine, Shimane University, Izumo, Japan.; ^2^ Community Health Nursing, Graduate School of Health Sciences, Okayama University, Okayama, Japan.; ^3^ Division of Environmental and Preventive Medicine, Faculty of Medicine, Tottori University, Yonago, Japan.

**Keywords:** problematic Internet use, child development, thinness, body mass index

## Abstract

This study aimed to clarify the association between mothers' problematic Internet use (PIU) and the thinness of their children. We analyzed data collected from health examinations of young children aged 4 months, 1.5 years, and 3 years of age performed in Matsue city, Japan, between April 2016 and March 2017. The subjects comprised 1,685 (866 boys, 819 girls) children aged 4 months, 1,728 (898 boys, 830 girls) aged 1.5 years, and 1,672 (802 boys, 870 girls) aged 3 years. Logistic regression analysis was used to clarify the association between mothers' PIU (Young's Diagnostic Questionnaire for Internet Addiction score: ≥4) and the thinness (body mass index: <15) of their children after adjusting for covariates such as birth weight, nutritional form, parental smoking status, maternal age, skipping breakfast, eating snacks, sleeping late, outdoor play, and daytime caregiver. Analysis after stratification by sex and age revealed that the mothers' PIU was significantly associated with their children's thinness only in boys aged 4 months or 1.5 years (odds ratio [OR] = 3.16, 95% confidence interval [CI] = 1.00–9.96 and OR = 2.68, 95% CI = 1.04–6.89, respectively). Mothers' PIU may promote thinness among boys aged <3 years. As the nutritional status of children aged <3 years is affected by maternal feeding attitudes, our findings suggested that mothers who exhibit PIU do not provide adequate care for their children, particularly regarding feeding. In contrast, no association between mothers' PIU and their children's thinness was observed in girls.

## Introduction

Internet access has rapidly increased in recent years, enhancing the quality of life related to communication, education, businesses, recreation, and many other aspects. On the contrary, there are concerns with users developing problems in interpersonal relationships and social life because of difficulty in controlling Internet use.^1^ Such a condition is known as “problematic Internet use (PIU)” and has drawn global attention since 1995 as a relatively new disorder.^2–4^

Several terms have replaced PIU such as “Internet addiction,” “Internet dependency,” “compulsive Internet use,” “pathological Internet use,” and “compulsive computer use.”^5^ This study consistently regarded “PIU” as a condition of being absorbed in the Internet and developing consequent problems in social life. Several investigators have reported that PIU may have many adverse consequences, including less than regular exercise, skipping meals, and late bedtimes,^6^ resulting in obesity and being overweight^7–9^ or underweight.^7^ However, all the aforementioned studies involved adolescents, and only a few studies have examined adult PIU,^10–12^ subsequently reporting an adult PIU prevalence rate of 4.0%–6.2%. Although the prevalence rate of adolescent PIU is 6.0%,^13^ which has been drawing attention, some researchers warn against underestimating adult PIU.^12^

The number of studies focusing on PIU among pregnant women and mothers is even smaller. Accordingly, Fujioka et al.^14^ examined mothers aged ≥20 years using the Young's Internet Addiction Test^15^ during health examinations for children aged 1.5 years, and reported that 2.8% of these mothers were suspected to be problematic users. In addition, they clarified that the level of Internet addiction increases with negative emotions related to parenting, represented by a sense of parenting burden and anxiety. In another previous study, the prevalence rates of PIU among women aged 16–29 and 30–39 years were 1.7% and 1.4%, respectively.^10^ Moreover, the rate among female university students who may become mothers in the near future has been markedly varied among studies, for example, from 4%^16^ to 28.7%,^17^ 31.7%,^18^ and 35.8%,^19^ indicating the necessity for further investigation of mothers' PIU.

Mothers' PIU may lead to inadequate parenting and carries the risk of interfering with the normal development of their children. Previous studies have explained this through consequent problems such as little sleep and failure to eat for long periods.^6,20^ Regarding PIU during parenting, there are concerns over the increased risk of neglect. Indeed, in a case study by Young,^1^ one mother was engrossed in the Internet to the extent that she neglected cooking, cleaning, shopping, and other household duties, consequently making her children feel neglected. Such a situation may even disrupt the family. In a study by Oka et al.,^21^ parental PIU led to sleep disorders in not only the parents themselves but also in their children, negatively influencing the latter's emotions and behaviors. Considering that PIU among mothers leads to insufficient parenting, such as neglect, their children may become thin in addition to developing sleep disorders. A previous study demonstrated that PIU leads to problematic eating habits associated with being underweight, including decreased dietary intake and/or appetite, and skipping dinner.^7^ Therefore, this study focused on children's body size as an index of their health conditions to examine the association between mothers' PIU and their children's thinness. The clarification of such an association may provide a basis for confirming whether the former leads to inappropriate parenting or even child abuse.

## Methods

### Study design and data sources

In 2017, a cross-sectional study was conducted in Matsue city, Shimane prefecture, to analyze data from health examinations of children aged 4 months, 1.5 years, and 3 years. Matsue city is a provincial city with a population size of ∼200,000 and birth rate of 1,700 births per year. The data were provided by Matsue city after integrating the mothers' responses to the Young's Diagnostic Questionnaire for Internet Addiction (YDQ)^22^ collected during the health examinations and their children's results using unlinkable anonymization. Accordingly, 1,733 children aged 4 months (participation rate: 99.5%), 1,798 aged 1.5 years (98.3%), and 1,754 aged 3 years (97.9%) were included.

The exclusion criteria included the following: multiple births that increase the risks of malnutrition and being underweight^23^ (4 months: 22, 1.5 years: 26, and 3 years: 28), extremely low birth weight (<1,000 g) that may lead to underdevelopment^24^ (1.5 years: 1 and 3 years: 2), child abuse because of the mother's own mental disorder/developmental disability or infant home use because of parenting difficulty (4 months: 10 and 1.5 years: 9), and YDQ respondents other than mothers (4 months: 16, 1.5 years: 34, and 3 years: 52). As children with severe diseases or disabilities are managed by medical institutions, they did not participate in health examinations. Consequently, 1,685 (866 boys, 819 girls) children aged 4 months, 1,728 (898 boys, 830 girls) aged 1.5 years, and 1,672 (802 boys, 870 girls) aged 3 years were included in the study.

Matsue city provided the data after removing personal identification information, including the name, address, and birth date, and supplementing ID numbers. This study was approved by the Ethics Committee of the School of Medicine, Shimane University (approval number: 2519).

### Measurements

Thinness among children was assessed using the body mass index (BMI) (weight [kg]/height [m]^2^). As many countries have individually created a BMI chart for each age group, the cutoffs vary.^25^ Using the standard body size of children based on a BMI of 15–19^26^ in Japan, this study defined thinness as a BMI of <15.

PIU was assessed using the YDQ,^22^ a scale consisting of eight questions regarding the pathological gambling criteria defined in the *Diagnostic and Statistical Manual, 4th Edition* (*DSM-IV*) that are answered with <Yes/No>. We used the YDQ in this study because it is one of the most widely used PIU assessment scales.^2,3,13,27,28^ Furthermore, it has previously been used in surveys involving adult women,^10^ and demonstrated to have acceptable reliability and consistency, as represented by a Spearman–Brown coefficient of 0.729 for split-half reliability, Cronbach's alpha of 0.713 with a standardized item alpha of 0.759,^29^ and Cronbach's alpha of 0.789.^30^ Based on this scale, respondents who answered <Yes> to five or more of the eight questions were classified as addicted Internet users. Moreover, to screen for at-risk Internet users, YDQ scores of 3–4 were used as a criterion.^10,29^ Only those who answered all the YDQ questions in this study were analyzed. The Cronbach's alpha of the YDQ in our study was 0.546–0.645.

### Covariates

Previous studies have found that birth weight,^31–33^ maternal age,^31^ parental smoking,^34,35^ and financial status^33,36,37^ are mainly associated with thinness in children. Factors associated with their body size included nutritional form,^38,39^ daytime caregiver,^40,41^ breakfast,^42,43^ duration of sleep,^44^ snacks,^43,44^ and physical activity.^44,45^

Based on the aforementioned findings, the following items were used as covariates to analyze children aged 4 months: birth weight (low birth weight: <2,500 g or others: ≥2,500 g), nutritional form (breastfeeding only, formula feeding, or breast and formula feeding), mother's current smoking status (nonsmoking or smoking), father's current smoking status (nonsmoking or smoking), maternal age (normal: 20–34 years, elderly: ≥35 years, or young: ≤19 years), and daytime caregiver (mother or others). Although parental smoking itself is associated with children's body size, this study used this variable considering that it also reflects the parental socioeconomic status because smoking rates are higher among individuals in lower socioeconomic positions.^46^ For children aged 1.5 and 3 years, skipping breakfast, eating snacks, sleeping late, and playing outdoors were also used in addition to the covariates used for those aged 4 months, excluding nutritional form. Multivariate analysis confirmed the multicollinearity among these variables.

### Statistical analysis

The children's characteristics were examined by calculating their numbers and proportions based on age and sex. The χ^2^ test was conducted for each sex to clarify sex differences among somatotypes and associated factors.

Subsequently, the association between mothers' PIU and thinness of their children was examined based on sex and age in months. As only ∼1% had a YDQ score ≥5, the cutoff was set at 4. Logistic regression analysis was performed with children's thinness (BMI <15) as the dependent variable and mothers' PIU (YDQ ≥4) as the explanatory variable while incorporating the aforementioned covariates. Univariate logistic regression analysis was initially performed, followed by multivariate logistic regression analysis for two models: Model 1 incorporating birth weight and nutritional form (only the former for children aged 1.5 and 3 years) and Model 2 incorporating mother's current smoking status, father's current smoking status, maternal age, daytime caregiver, breakfast, bedtime, snacks, outdoor play, and daytime caregiver (only the current smoking status of the mother and father, maternal age, and daytime caregiver were incorporated for children aged 4 months).

Sensitivity was analyzed using the data, assigning a score of 0 to questions without answers, to confirm changes in the results.

IBM SPSS Statistics 22 was used for the analysis, with the significance level set at <5%.

## Results

The characteristics of the children aged 4 months, 1.5 years, and 3 years are given in Table 1. The proportions of children with BMI <15 among children aged 4 months, 1.5 years, and 3 years were 7.3%, 14.6%, and 29.4%, respectively. Significant sex differences were found in BMI values for children aged 4 months or 1.5 years. Moreover, 2.8%, 3.2%, and 3.1% of mothers with children aged 4 months, 1.5 years, and 3 years had a YDQ score of ≥4, respectively.

**Table 1.** Characteristics of Children

	*4 Months old (*n* = 1,685)*				*1 Year and 6 months old (*n* = 1,728)*				*3 Years old (*n* = 1,672)*			
	*Total* n *(%)*	*Boys* n *(%)*	*Girls* n *(%)*	p	*Total* n *(%)*	*Boys* n *(%)*	*Girls* n *(%)*	p	*Total* n *(%)*	*Boys* n *(%)*	*Girls* n *(%)*	p
Birth weight (*n* = 1,679)					(*n* = 1,724)				(*n* = 1,208)			
Low birth weight (<2,500)	117 (6.9)	50 (5.8)	67 (8.2)	0.052	143 (8.3)	49 (5.5)	94 (11.3)	<0.001	83 (5.0)	36 (4.5)	47 (5.4)	0.510
Others (≥2,500)	1,562 (92.7)	813 (93.9)	749 (91.5)		1,581 (91.5)	846 (94.2)	735 (88.6)		1,125 (67.3)	530 (66.1)	595 (68.4)	
Nutritional form (*n* = 1,670)												
Exclusive breast feeding	1,104 (65.5)	557 (64.3)	547 (66.8)	0.324								
Formula feeding or breast and formula feeding	566 (33.6)	300 (34.6)	266 (32.5)									
BMI (*n* = 1,685)					(*n* = 1,728)				(*n* = 1,668)			
Thinness (<15)	123 (7.3)	48 (5.5)	75 (9.2)	0.004	252 (14.6)	101 (11.2)	151 (18.2)	<0.001	491 (29.4)	222 (27.7)	269 (30.9)	0.138
Others (≥15)	1,562 (92.7)	818 (94.5)	744 (90.8)		1,476 (85.4)	797 (88.8)	679 (81.8)		1,177 (70.4)	579 (72.2)	598 (68.7)	
Mother's smoking status (*n* = 1,672)					(*n* = 1,723)				(*n* = 1,631)			
Smoking	37 (2.2)	19 (2.2)	18 (2.2)	0.98	65 (3.8)	32 (3.6)	33 (4.0)	0.648	89 (5.3)	43 (5.4)	46 (5.3)	0.924
Nonsmoking	1,635 (97.0)	843 (97.3)	792 (96.7)		1,658 (95.9)	864 (96.2)	794 (95.7)		1,542 (92.2)	737 (91.9)	805 (92.5)	
Father's smoking status (*n* = 1,665)					(*n* = 1,723)				(*n* = 1,631)			
Smoking	566 (33.6)	287 (33.1)	279 (34.1)	0.629	606 (35.1)	334 (37.2)	272 (32.8)	0.057	538 (32.2)	262 (32.7)	276 (31.7)	0.619
Nonsmoking	1,099 (65.2)	571 (65.9)	528 (64.5)		1,117 (64.6)	562 (62.6)	555 (66.9)		1,093 (65.4)	518 (64.6)	575 (66.1)	
Maternal age (*n* = 1,678)					(*n* = 1,726)				(*n* = 1,668)			
Elderly (≥35)	481 (28.5)	257 (29.7)	224 (27.4)	[0.299 [0.563	379 (21.9)	200 (22.3)	179 (21.6)	[0.724 [0.400	396 (23.7)	194 (24.2)	202 (23.2)	[0.659 [0.890
Normal (20–34)	1,177 (69.9)	597 (68.9)	580 (70.8)		1,326 (76.7)	688 (76.6)	638 (76.9)		1,260 (75.4)	601 (74.9)	659 (75.7)	
Young (≤19)	20 (1.2)	9 (1.0)	11 (1.3)		21 (1.2)	9 (1.0)	12 (1.4)		12 (0.7)	6 (0.7)	6 (0.7)	
Mother's YDQ score (*n* = 1,630)					(*n* = 1,667)				(*n* = 1,608)			
High (≥5)	18 (1.1)	12 (1.4)	6 (0.7)	0.195	25 (1.4)	11 (1.2)	14 (1.7)	0.412	17 (1.0)	5 (0.6)	12 (1.4)	0.122
Low (<5)	1,612 (95.7)	827 (95.5)	785 (95.8)		1,642 (95.0)	858 (95.5)	784 (94.5)		1,591 (95.2)	768 (95.8)	823 (94.6)	
High (≥4)	47 (2.8)	29 (3.5)	18 (2.2)	0.154	55 (3.2)	26 (2.9)	29 (3.5)	0.463	51 (3.1)	22 (2.7)	29 (3.3)	0.473
Low (<4)	1,583 (93.9)	810 (96.5)	773 (94.4)		1,612 (93.3)	843 (93.9)	769 (92.7)		1,557 (93.1)	751 (93.6)	806 (92.6)	
Day caretaker (*n* = 1,674)					(*n* = 1,722)				(*n* = 1,665)			
Mother	1,571 (93.2)	804 (92.8)	767 (93.7)	0.164	507 (29.3)	272 (30.3)	235 (28.3)	0.403	230 (13.8)	110 (13.7)	120 (13.8)	0.995
Except mother	103 (6.1)	60 (6.9)	43 (5.3)		1,215 (70.3)	625 (69.6)	590 (71.1)		1,435 (85.8)	686 (85.5)	749 (86.1)	
Skipping breakfast					(*n* = 1,715)				(*n* = 1,661)			
Skipping					52 (3.0)	30 (3.3)	22 (2.7)	0.400	80 (4.8)	32 (4.0)	48 (5.5)	0.152
Eating					1,663 (96.2)	861 (95.9)	802 (96.6)		1,581 (94.6)	762 (95.0)	819 (94.1)	
Sleeping late					(*n* = 1,727)				(*n* = 1,670)			
After 10 p.m.					232 (13.4)	111 (12.4)	121 (14.6)	0.174	496 (29.7)	229 (28.6)	267 (30.7)	0.324
Before 10 p.m.					1,495 (86.5)	787 (87.6)	708 (85.3)		1,174 (70.2)	573 (71.4)	601 (69.1)	
Snack					(*n* = 1,707)				(*n* = 1,665)			
No snack					20 (1.2)	13 (1.4)	7 (0.8)	0.240	7 (0.4)	4 (0.5)	3 (0.3)	0.456
Eating snacks					1,687 (97.6)	874 (97.3)	813 (98.0)		1,658 (99.2)	795 (99.1)	863 (99.2)	
Outside play					(*n* = 1,704)				(*n* = 1,640)			
Inactivity					34 (2.0)	50 (5.6)	66 (8.0)	0.922	117 (7.0)	51 (6.4)	66 (7.6)	0.337
Activity					1,670 (96.6)	838 (93.3)	750 (90.4)		1,523 (91.1)	734 (91.5)	789 (90.7)	

YDQ, Young's Diagnostic Questionnaire for Internet Addiction.

As given in Tables 2–4, univariate logistic regression analysis found that the mothers' PIU was significantly correlated with their children's thinness in boys aged 4 months and 1.5 years (odds ratio [OR] = 2.85, 95% confidence interval [CI] = 0.95–8.57 and OR = 3.67, 95% CI = 1.55–8.69, respectively). However, no significant correlation was observed between thinness in boys aged 3 years old and their mothers' PIU (OR = 1.23, 95% CI = 0.50–3.07). Furthermore, no significant correlation was found between thinness in girls aged 4 months, 1.5 years, or 3 years and their mothers' PIU (OR = 1.23, 95% CI = 0.50–3.07; OR = 1.45, 95% CI = 0.61–3.47; and OR = 1.37, 95% CI = 0.64–2.94, respectively). The multivariate logistic regression analysis provided similar results. The mothers' PIU was found to be significantly correlated with their children's thinness in boys aged 4 months or 1.5 years (OR = 3.16, 95% CI = 1.00–9.96 and OR = 2.68, 95% CI = 1.04–6.89, respectively). However, no significant correlation was found between thinness in boys aged 3 years and their mothers' PIU (OR = 0.66, 95% CI = 0.14–3.20). Furthermore, no significant correlation was observed between thinness in girls aged 4 months, 1.5 years, and 3 years and their mothers' PIU (OR = 0.58, 95% CI = 0.07–4.55; OR = 1.27, 95% CI = 0.51–3.18; and OR = 1.25, 95% CI = 0.48–3.24, respectively). The sensitivity analysis yielded similar results.

**Table 2.** Adjusted Risk Ratio with 95% Confidence Interval for the Association Between Mothers' Problematic Internet Use and Thinness (Body Mass Index <15) in Children Aged 4 Months

	*Boys*						*Girls*					
	*Crude*		*Model 1*		*Model 2*		*Crude*		*Model 1*		*Model 2*	
	*OR (95% CI)*	p	*OR (95% CI)*	p	*OR (95% CI)*	p	*OR (95% CI)*	p	*OR (95% CI)*	p	*OR (95% CI)*	p
YDQ												
Low (<4)	Ref		Ref		Ref		Ref		Ref		Ref	
High (≥4)	2.85 (0.95–8.57)	0.062	2.97 (0.96–9.20)	0.059	3.16 (1.00–9.96)	0.049	1.23 (0.50–3.07)	0.650	0.59 (0.08–4.58)	0.618	0.58 (0.07–4.55)	0.607
Birth weight												
Others (≥2,500)	Ref		Ref		Ref		Ref		Ref		Ref	
Low birth weight (<2,500)	3.07 (1.30–7.23)	0.011	2.98 (1.24–7.19)	0.015	2.86 (1.18–6.93)	0.020	1.40 (0.64–3.06)	0.395	1.36 (0.62–3.00)	0.443	1.21 (0.52–2.79)	0.657
Nutritional form												
Exclusive breast feeding	Ref		Ref		Ref		Ref		Ref		Ref	
Formula feeding or breast and formula feeding	2.52 (1.40–4.55)	0.002	2.68 (1.47–4.90)	0.001	3.03 (1.62–5.67)	0.001	1.92 (1.19–3.10)	0.008	1.88 (1.14–3.08)	0.013	1.98 (1.18–3.33)	0.009
Mother's smoking status												
Nonsmoking	Ref				Ref		Ref				Ref	
Smoking	NA	—			NA	—	0.59 (0.08–4.48)	0.609			0.66 (0.08–5.39)	0.694
Father's smoking status												
Nonsmoking	Ref				Ref		Ref				Ref	
Smoking	1.10 (0.60–2.02)	0.766			1.28 (0.68–2.41)	0.452	0.98 (0.59–1.63)	0.951			0.93 (0.54–1.60)	0.793
Maternal age												
Normal (20–34)	Ref				Ref		Ref				Ref	
Elderly (≥35)	1.29 (0.70–2.38)	0.409			1.17 (0.62–2.22)	0.624	0.92 (0.53–1.60)	0.770			0.78 (0.44–1.40)	0.408
Young (≤19)	NA	—			NA	—	2.21 (0.47–10.49)	0.319			1.02 (0.12–8.61)	0.984
Day caretaker												
Mother	Ref				Ref		Ref				Ref	
Other than mother	0.89 (0.27–2.95)	0.846			0.56 (0.16–1.97)	0.367	0.74 (0.22–2.44)	0.615			0.41 (0.09–1.82)	0.242

CI, confidence interval; Model 1, adjusted for birth weight and nutritional form; Model 2, Model 1 + adjusted for mother's smoking status, father's smoking status, maternal age, and day caretaker; NA, not available; OR, odds ratio; Ref, reference.

**Table 3.** Adjusted Risk Ratio with 95% Confidence Interval for the Association Between Mothers' Problematic Internet Use and Thinness (Body Mass Index <15) in Children Aged 1 Year and 6 Months

	*Boys*						*Girls*					
	*Crude*		*Model 1*		*Model 2*		*Crude*		*Model 1*		*Model 2*	
	*OR (95% CI)*	p	*OR (95% CI)*	p	*OR (95% CI)*	p	*OR (95% CI)*	p	*OR (95% CI)*	p	*OR (95% CI)*	p
YDQ												
Low (<4)	Ref		Ref		Ref		Ref		Ref		Ref	
High (≥4)	3.67 (1.55–8.69)	0.003	3.46 (1.44–8.33)	0.006	2.68 (1.04–6.89)	0.041	1.45 (0.61–3.47)	0.398	1.49 (0.62–3.56)	0.371	1.27 (0.51–3.18)	0.604
Birth weight												
Others (≥2,500)	Ref		Ref		Ref		Ref		Ref		Ref	
Low birth weight (<2,500)	3.53 (1.83–6.83)	<0.001	3.37 (1.69–6.73)	0.001	3.41 (1.65–7.03)	0.001	1.35 (0.80–2.28)	0.257	1.35 (0.79–2.31)	0.267	1.44 (0.81–2.53)	0.213
Mother's smoking status												
Nonsmoking	Ref				Ref		Ref				Ref	
Smoking	1.14 (0.39–3.33)	0.807			1.41 (0.46–4.35)	0.553	1.47 (0.65–3.32)	0.356			1.21 (0.47–3.13)	0.700
Father's smoking status												
Nonsmoking	Ref				Ref		Ref				Ref	
Smoking	0.59 (0.37–0.94)	0.025			0.63 (0.38–1.03)	0.065	1.18 (0.82–1.72)	0.371			1.23 (0.82–1.84)	0.315
Maternal age												
Normal (20–34)	Ref				Ref		Ref				Ref	
Elderly (≥35)	0.72 (0.42–1.23)	0.231			0.69 (0.39–1.22)	0.203	0.94 (0.61–1.46)	0.790			0.89 (0.56–1.42)	0.621
Young (≤19)	NA	—			NA	—	2.25 (0.67–7.60)	0.192			1.92 (0.52–7.06)	0.325
Skipping of breakfast												
Eating	Ref				Ref		Ref				Ref	
Skipping	2.06 (0.82–5.18)	0.123			1.92 (0.73–5.10)	0.188	1.00 (0.33–2.99)	0.998			1.02 (0.32–3.25)	0.973
Bedtime												
Before 10 p.m.	Ref				Ref		Ref				Ref	
After 10 p.m.	1.78 (1.03–3.06)	0.039			1.60 (0.88–2.90)	0.125	1.60 (1.01–2.53)	0.044			1.17 (0.69–2.00)	0.558
Snack												
Eat snacks	Ref				Ref		Ref				Ref	
No snack	1.47 (0.32–6.75)	0.618			0.98 (0.20–4.74)	0.981	1.78 (0.34–9.28)	0.492			1.07 (0.18–6.24)	0.939
Outside play												
Activity	Ref				Ref		Ref				Ref	
Inactivity	1.08 (0.45–2.60)	0.865			0.78 (0.29–2.11)	0.626	1.62 (0.91–2.91)	0.103			1.12 (0.57–2.22)	0.734
Day caretaker												
Mother	Ref				Ref		Ref				Ref	
Other than mother	0.47 (0.31–0.72)	0.001			0.49 (0.31–0.77)	0.002	0.34 (0.24–0.50)	<0.001			0.34 (0.23–0.50)	<0.001

Model 1, adjusted for birth weight; Model 2, Model 1 + adjusted for mother's smoking status, father's smoking status, maternal age, skipping breakfast, bedtime, snack, outside play, and day caretaker.

**Table 4.** Adjusted Risk Ratio with 95% Confidence Interval for the Association Between Mothers' Problematic Internet Use and Thinness (Body Mass Index <15) in Children Aged 3 Years

	*Boys*						*Girls*					
	*Crude*		*Model 1*		*Model 2*		*Crude*		*Model 1*		*Model 2*	
	*OR (95% CI)*	p	*OR (95% CI)*	p	*OR (95% CI)*	p	*OR (95% CI)*	p	*OR (95% CI)*	p	*OR (95% CI)*	p
YDQ												
Low (<4)	Ref		Ref		Ref	Ref	Ref		Ref		Ref	
High (≥4)	1.23 (0.50–3.07)	0.650	0.57 (0.12–2.68)	0.479	0.66 (0.14–3.20)	0.609	1.37 (0.64–2.94)	0.422	1.25 (0.49–3.21)	0.643	1.25 (0.48–3.24)	0.649
Birth weight												
Others (≥2,500)	Ref		Ref		Ref	Ref	Ref		Ref		Ref	
Low birth weight (<2,500)	1.86 (0.93–3.70)	0.079	1.84 (0.92–3.67)	0.084	2.04 (0.96–4.32)	0.064	2.01 (1.10–3.67)	0.023	2.19 (1.18–4.04)	0.012	2.06 (1.09–3.89)	0.027
Mother's smoking status												
Nonsmoking	Ref				Ref		Ref				Ref	
Smoking	1.16 (0.59–2.28)	0.660			0.77 (0.29–2.02)	0.592	1.58 (0.86–2.90)	0.137			1.35 (0.55–3.30)	0.516
Father's smoking status												
Nonsmoking	Ref				Ref		Ref				Ref	
Smoking	1.25 (0.90–1.74)	0.179			1.51 (0.99–2.30)	0.054	0.90 (0.66–1.23)	0.511			0.94 (0.63–1.40)	0.774
Maternal age												
Normal (20–34)	Ref				Ref		Ref				Ref	
Elderly (≥35)	1.02 (0.71–1.46)	0.928			1.03 (0.65–1.61)	0.911	1.23 (0.88–1.72)	0.218			1.32 (0.87–2.01)	0.191
Young (≤19)	1.32 (0.24–7.26)	0.751			1.64 (0.13–20.05)	0.697	1.11 (0.20–6.08)	0.907			1.47 (0.17–12.45)	0.722
Skipping of breakfast												
Eating	Ref				Ref		Ref				Ref	
Skipping	1.38 (0.65–2.90)	0.401			1.11 (0.46–2.67)	0.812	1.78 (0.99–3.21)	0.055			2.19 (1.01–4.74)	0.046
Bedtime												
Before 10 p.m.	Ref				Ref		Ref				Ref	
After 10 p.m.	1.23 (0.88–1.72)	0.234			1.19 (0.76–1.87)	0.439	1.22 (0.90–1.66)	0.206			1.04 (0.70–1.54)	0.859
Snack												
Eat snacks	Ref				Ref		Ref				Ref	
No snack	NA	—			NA	—	1.11 (0.10–12.30)	0.932			1.85 (0.11–31.75)	0.671
Outside play												
Activity	Ref				Ref		Ref				Ref	
Inactivity	1.22 (0.66–2.25)	0.528			0.89 (0.36–2.21)	0.797	1.12 (0.66–1.91)	0.684			0.70 (0.33–1.47)	0.349
Day caretaker												
Mother	Ref				Ref		Ref				Ref	
Other than mother	0.94 (0.60–1.48)	0.797			0.79 (0.44–1.41)	0.423	0.56 (0.38–0.83)	0.004			0.50 (0.29–0.87)	0.013

Model 1, adjusted for birth weight; Model 2, Model 1 + adjusted for mother's smoking status, father's smoking status, maternal age, skipping breakfast, bedtime, snack, outside play, and day caretaker.

## Discussion

Our study demonstrated an association between mothers' PIU and the thinness of their children. Although many previous studies focused on adolescent PIU,^12,47^ and mainly examined the prevalence of PIU, diagnostic criteria for PIU, the association between PIU and mental health, and risk factors for PIU,^47–49^ this study may be important in that it clarified not only the prevalence of PIU among mothers, but also problems because of their PIU. Among mothers rearing children, those with a YDQ score ≥5 or 4 accounted for ∼1% and 3% of the total subjects, respectively. Fujioka et al.^14^ using YIAT20^15^ found that PIUs accounted for 2.8%, which is close to the rate of a YDQ score ≥4 in our study. Similar values were also reported by Bakken et al.^10^ using the YDQ,^22^ with women having a YDQ score of ≥5 or 3–4, accounting for 1.4% (aged 30–39 years) – 1.7% (aged 16–29 years) and 4.8%–13.1%, respectively. Thus, a PIU prevalence rate of 1%–3% among mothers may be a useful criterion for future studies.

The prevalence of thinness was 3.16 and 2.68 times higher among boys aged 4 months and 1.5 years, respectively, when their mothers had a YDQ score of ≥4. Children aged 4 months are within the lactation period, whereas children aged 1.5 years cannot independently ingest nutrients. Therefore, thinness in such children may be because of insufficient nutrient intake as a result of poor parenting by their mothers who are absorbed in the Internet. Moreover, several previous studies revealed the association between adolescent PIU and skipping meals^6,20^ and decreased dietary intake and/or appetite, which can lead to being underweight.^7^ This suggests that mothers with PIU are thin because of insufficient dietary intake, and as thinness in parents is associated with thinness in their children,^50,51^ there may be an association between mothers' PIU and thinness in their children. As parental PIU has been reported to cause sleep disorders in both parents and their children,^21^ this study may have significance in suggesting the direct and indirect negative influences of PIU on the users themselves and those around them, respectively.

Furthermore, the association between mothers' PIU and thinness was only observed in boys and not in girls. This may be explained by higher total energy expenditure^52–54^ and requirements^54^ for male infants and toddlers. Thus, the effects of insufficient nutrient and energy intakes may have consequently been more marked on boys than on girls, resulting in a higher prevalence of thinness among the boys. In contrast, there was no association between mothers' PIU and thinness in children aged 3 years regardless of sex. At this age, children are able to independently ingest nutrients and many go to nursery school. Moreover, the daytime caregiver was not the mother in 85.8% of the cases. Consequently, the influence of mothers' PIU on parenting may have been less marked.

This study had the following limitations. First, given the cross-sectional design of this study, causal relationships were unable to be clarified. Therefore, cohort studies should be conducted in the future to examine whether a similar tendency is observed in other populations by confirming the consistency and strength of association involving a larger number of samples, while considering the temporal relationship as an element of causal inference. However, thinness among children is highly unlikely to lead to PIU in their mothers, whereas maternal PIU may be more a reasonable cause of thinness in their children. Second, considering that mothers with a YDQ score of ≥4 accounted for ∼3% of the sample, such a small sample size may have resulted in beta errors. However, an association was found, suggesting further possibilities, for example, the same association in other age groups or an association between mothers' PIU and obesity. Third, PIU has been reported to be associated with mental disorders, such as attention-deficit/hyperactivity disorder (ADHD), depression, obsessive symptoms, and hostility,^28,55–58^ with several previous studies observing a particularly close association between PIU and ADHD.^28,57^ Although it was difficult to exclude mothers with ADHD in our study, the analysis was performed without those using infant homes, suggesting that mothers with parenting difficulties because of severe mental symptoms were excluded. Fourth, we did not use the International Obesity Task Force thinness grades^59^ or World Health Organization *z*-scores (< −2 *SD*),^37,60^ which are international criteria for thinness among children, thereby making international comparisons difficult. However, as measurement of the height of children younger than 2 years is prone to errors, screening was performed for those exhibiting a tendency toward thinness, which requires intervention to the extent that clinical significance may be achieved by assessing the condition using standard reference values.

Although such limitations were present, the importance of this study is that it suggests that mothers' PIU indirectly leads to thinness in their children. Internet use by pregnant women and mothers has been demonstrated to be a useful method to collect health information and develop appropriate health-related behaviors^61–65^; however, this study hypothesizes that mothers' PIU increases the risk of health impairment in their children. In particular, the thinness of children was reported to be associated with increased risks of infectious disease, poorer cognitive performance, and/or lower school grades, and is a risk factor for diabetes and cardiovascular disease during adulthood.^66^ The provision of health guidance for mothers to appropriately use the Internet may be effective for promoting the healthy growth and development of their children. As future research perspectives, it may be necessary to further clarify the association between mothers' PIU and their children's injuries, dental caries, or other health problems, and the influence of mothers' PIU on the children's health when they become adolescents to confirm whether mothers' PIU increases the risk of health problems in their children. Based on the results of these future studies, it may be possible to establish a system to screen for mothers' PIU during health examinations for infants, to explain to mothers with PIU that their condition may negatively affect their children's growth and development, and to caution them about excessive Internet use.

## Conclusions

Mothers' PIU was associated with thinness in boys aged 4 months or 1.5 years. To understand this finding further, the limitations of this study need to be resolved. Moreover, future surveys should examine the association between mothers' PIU and their children's body size in more detail.
